# The FnBPA from methicillin-resistant *Staphylococcus aureus* promoted development of oral squamous cell carcinoma

**DOI:** 10.1080/20002297.2022.2098644

**Published:** 2022-07-15

**Authors:** Li-Xin Kong, Zheng Wang, Yu-Ke Shou, Xue-Dong Zhou, Ya-Wen Zong, Ting Tong, Min Liao, Qi Han, Yan Li, Lei Cheng, Biao Ren

**Affiliations:** a State Key Laboratory of Oral Diseases & National Clinical Research Center for Oral Diseases & West China Hospital of Stomatology, Sichuan University, Chengdu, Sichuan, China; b Department of Operative Dentistry and Endodontics, West China School of Stomatology, Sichuan University, Chengdu, Sichuan, China

**Keywords:** Drug resistance, cell cycle, fibronectins, cell proliferation, proto-oncogene proteins c-fos

## Abstract

**Background:**

Oral squamous cell carcinoma (OSCC) is the most common tumor in the oral cavity. Methicillin-resistant *Staphylococcus aureus* (MRSA) were highly detected in OSCC patients; however, the interactions and mechanisms between drug-resistant bacteria (MRSA) and OSCC are not clear.

**Aim:**

The aim of this study was to investigate the promotion of MRSA on the development of OSCC.

**Methods:**

MRSA and MSSA (methicillin-susceptible) strains were employed to investigate the effect on the proliferation of OSCC in *vitro* and *vivo*.

**Results:**

All of the MRSA strains significantly increased the proliferation of OSCC cells and MRSA arrested the cell cycles of OSCC cells in the S phase. MRSA activated the expression of TLR-4, NF-κB and c-fos in OSCC cells. MRSA also promoted the development of squamous cell carcinoma in vivo. The virulence factor *fnbpA* gene was significantly upregulated in all MRSA strains. By neutralizing FnBPA, the promotions of MRSA on OSCC cell proliferation and development of squamous cell carcinoma were significantly decreased. Meanwhile, the activation of c-fos and NF-κB by MRSA was also significantly decreased by FnBPA antibody.

**Conclusion:**

MRSA promoted development of OSCC, and the FnBPA protein was the critical virulence factor. Targeting virulence factors is a new method to block the interaction between a drug-resistant pathogen and development of tumors.

## Introduction

Oropharyngeal cancer is the sixth most common malignant tumor in the world with the incidence of more than 500,000 annually [1,2]. Oral squamous cell carcinoma (OSCC), the most common oral cancer [3], is highly invasive and prone to lymph node or distant organ metastasis [4], such as lung and heart [5]. The 5-year survival rate of OSCC was even less than 60% [6]. Dysbiosis of the microbiome and some key microorganisms are considered key risk factors [7].

*Staphylococcus aureus* is one of the most common oral and perioral bacteria [8]. In the oral cancer patients after radiotherapy and chemotherapy, *S. aureus* became the dominant bacteria in blood and the oral cavity [9]. The lipoteichoic acid of *S. aureus* can promote the proliferation of lung cancer cells [10], while the enterotoxin C1 of *S. aureus* can inhibit the growth of bladder cancer [11], indicating the strong relationship between *S. aureus* and cancer. Our previous study found that *S. aureus* can upregulate the *fnbpB* gene to activate Cyclooxygenase-2 (COX-2)/prostaglandin E2 (PGE2) pathway of oral epithelial cells to promote the malignant transformation of oral mucosal cells [12,13]. However, the mechanisms of *S. aureus* in the development of OSCC are not clear.

Due to the overuse of antibiotics, the infections caused by drug-resistant pathogens have become a global health challenge. Methicillin-resistant *S. aureus* (MRSA), first identified in 1961 [14], is one of the most common drug-resistant pathogens [15]. The MRSA infection increased the mortality nearly 50% and the detection rate of MRSA was high in cancer patients [16]. In OSCC patients, the detection rates of MRSA were about 75–77.8% [17–19]. According to the positive correlation between MRSA and cancers, MRSA was also considered as a key carcinogenic agent to take part in the occurrence and development of the tumor. However, the detailed mechanisms of MRSA acting on cancer are unknown.

Bacterial fibronectin-binding proteins (FnBPs) can bind to the fibronectin (FN) of host cells to regulate the response of the host to bacteria [20]. *S. aureus* has two main FnBPs, FnBPA and FnBPB, encoded by two closely linked genes *fnbpA* and *fnbpB*, respectively [21]. The *fnbpA* is more likely to be highly expressed in MRSA, while *fnbpB* is more likely to be expressed in MSSA (methicillin-susceptible) [22]. Both FnBPA and FnBPB can bind to the FN protein of host cells to promote the adhension of *S. aureus* to host cells, then the adherent bacteria can activate the transmembrane pattern recognition receptors of the host cells, such as TLR-4, a key receptor involved in recognition of pathogens [23–25]. The Toll-Like Receptor 4 (TLR4)/Nuclear Factor kappa-B (NF-κB)/mitogen-activated protein kinase (MAPK) signaling pathway was also activated in a variety of cancer cells and highly related to the occurrence and development of cancer [26,27]. However, the effects of MRSA, especially the FnBPs, on OSCC cells are not clear.

Therefore, we investigated the effects and possible mechanisms of MRSA on the development of OSCC *in vitro* and *in vivo*, to highlight the importance of oral bacteria in cancer development and the candidate anti-tumor target that addresses the bacteria–host interactions.

## Materials and methods

### Cell line, bacterial strains and culture

The cell lines of human OSCC Cal27 and SCC25 were cultured in high glucose Dulbecco’s Modified Eagle Medium (DMEM, Hyclone, Logan, UT) with 10% fetal bovine serum (FBS, Gibco, Thermo Fisher Scientific, Inc., Waltham, MA), 1% penicillin–streptomycin antibiotic mixture (PS, Hyclone, Logan, UT) and incubated at 37°C with 5% CO_2_ and 95% air. Cells were passaged at regular intervals depending on their growth characteristics using 0.25% trypsin (Hyclone, Logan, UT).

*S. aureus* strains ATCC 25923, ATCC 8325.4, ATCC 29213, ATCC 6538, No. 18908, No. 18466, No. 19423, No. 19047, No. 19494, No. 18878, No. 18858, No. 19041, No. 19900, No. 18567, No. 18475, No. 19167 and No. 19498 were stored in the State Key Laboratory of Oral Diseases and routinely cultured in Tryptone soya broth (TSB, Oxoid, Basingstoke, UK).

For the collection of *S. aureus* culture supernatant, *S. aureus* was cultured overnight at 37°C in 5 ml TSB medium and then diluted in fresh TSB medium at 1:5. After another incubation for 2 h, the culture was centrifuged at 4,000 × *g* for 10 min and the supernatant was discarded. The bacterial pellets were washed twice with sterilized PBS, then resuspended in DMEM high sugar medium (containing 10% FBS but without PS) at a concentration of 1 × 10^8^ CFU/ml. The suspension was then incubated in 37°C for 2 h. The culture was filtrated immediately and the supernatant was collected. The DMEM high glucose medium without *S. aureus* (containing 10% FBS but without double antibody) was incubated for 2 h under the same conditions, and the supernatant was filtered as the control group [28].

### MIC Test

The minimum inhibitory concentration (MIC) of *S. aureus* was determined by the twofold double dilution method as described previously [29]. MH culture medium, 100 µL, containing 256 μg/mL methicillin was added to the frst well in 96-well plates, and serial dilutions were made with 100 μL of MH liquid medium to obtain a series of concentration gradients. *S. aureus* was cultured overnight at 37°C in TSB medium and then diluted in fresh MH medium at a concentration of 1 × 10^6^ CFU/ml, then 100 µL of bacterial suspension was added to each well containing different concentrations of methicillin. After incubation at 37°C anaerobically for 24 h, the lowest concentration at which there was no visible bacterial growth was recorded as the MIC value. All experiments were done in triplicate.

### Cell proliferation measurement

In a 96-well plate, 2 × 10^3^ cells were incubated overnight. The medium was removed and replaced with the filtrated supernatant of *S. aureus* culture. After incubation for 24 h, cells were washed with PBS and the viable cells were measured using a CCK-8 assay kit (Dojindo) according to the manufacturer’s instructions. The absorbance was measured at 450 nm using a microplate reader (Thermo Fisher Scientific Inc., Waltham, MA). The OD values of each well were measured to represent the proliferation of cells [12]. Then, the proliferation rate of each group was calculated according to the following formula: cell proliferation rate (%) = (OD _experimental group_ – OD _control group_)/(OD _control group_- OD _blank control group_) × 100% [30]. All experiments were performed at least in triplicate.

### Cell cycle detection

In the present study, ATCC 8325.4 and No. 18466 were randomly selected as the represented strains of MSSA and MRSA. In a 6 cm culture dish, 8 × 10^4^ Cal27 cells were incubated overnight. The *S. aureus* filtrated supernatant was added. After 24 h, cell cycle staining was carried out by using a cell cycle staining kit (KeyGen Biotech, China) following the manufacturer’s instructions. DNA contents were analyzed using an Elite ESP flow cytometry (Beckman Coulter, CA) [31].

### RNA extraction and quantitative real‐time PCR (qRT‐PCR)

Total RNA of the Cal27 cells was isolated following the instructions of TRIzol reagent (Invitrogen, CA) after treatment with the *S. aureus* filtrated supernatant for 6 h. The cDNA was then synthesized by using a PrimeScript RT reagent kit with gDNA Eraser (Takara Clontech, Japan) according to the manufacturer’s instructions. Real-time PCR was performed on a C1000 Touch™ thermal cycler instrument (Bio-Rad, Philadelphia, PA) with SYBR reagent (Takara, Dalian, China) following the manufacturer’s instructions. Amplification was performed following a two-step strategy: (1) 94°C for 30 s; (2) 40 PCR cycles (94°C for 30 s, a gene-specific annealing temperature for 30 s). All primer sequences are listed in Table S1. The gene expression level relative to the calibrator was expressed as 2^−ΔΔCT^ [32].

For the preparation of *S. aureus* RNA, the bacterial culture was centrifugated at 4°C, and the bacterial pellets were flash frozen in liquid nitrogen and stored at −80°C until RNA preparation. Total RNA was isolated following the instructions provided with TRIzol reagent (Invitrogen, CA). The remaining RNA extraction methods, reverse transcription and real-time fluorescent quantitative PCR of bacterial mRNA expression levels are described as above [33]. The gene expression level relative to the calibrator was expressed as 2^−ΔΔCT^. All primer sequences are listed in Table S1.

### Western blotting

Frozen Cal27 cells were lysed in RIPA buffer containing protease inhibitor cocktail (Roche, Mannheim, Germany) for 30 min on ice and were then removed by centrifuging at 12,000 × *g* for 5 min. Proteins were quantified by the BCA method, separated by SDS-PAGE, transferred to nitrocellulose membranes, and analyzed with Western blotting. The membranes were probed with primary antibodies against β-actin, c-Fos at 4°C overnight. After incubation with HRP-conjugated polyclonal antibodies, the protein bands were visualized using an enhanced chemiluminescent substrate [12].

### Neutralization of FnBPA

*S. aureus* was cultured overnight at 37°C in TSB medium and then diluted in fresh TSB medium at 1:5 to incubate at 37°C for 2 h. Then the bacteria were harvested by centrifugation (4,000 *g*, 4°C, 10 min), washed with phosphate buffer saline and re-suspended (1 × 10^8^ CFU/ml) in high glucose DMEM Medium with 10% FBS. The FnBPA antibodies (Abnova, China) at different dosages (0, 0.25, 1 μg/mL) were added into the *S. aureus* suspension to neutralize the FnBPA protein and incubated at 37°C for 2 h. Then. the filtrated supernatants were used to treat the cells.

### Subcutaneous tumorigenicity assays

Experimental protocols followed ARRIVE guidelines 2.0: Updated guidelines for reporting animal research [34]. The study was conducted according to Institutional Animal Care and Use Committee policies and approved by the West China School of Stomatology Institute Review Board (WCSHIRB) ethics committee (WCHSIRB-D-2017-196). BALB/c nude mice were purchased from GemPharmatech Co., Ltd and fed in laminar flow cabinets under specific pathogen‐free conditions. The subcutaneous oral tumor model was established in nude mice (female, 4–6 weeks old). The Cal27 cells (2 × 10^6^ cells) in 0.1 ml culture medium were injected subcutaneously into the right flank of the nude mice. After 1 week of tumor formation, 15 mice were randomly divided into three groups: control, MSSA and MRSA, respectively. For the FnBPA neutralization treatment, 40 mice were randomly divided into five groups: control, MSSA, MRSA, MRSA+0.5 µl FnBPA antibody, MRSA+2 μl FnBPA antibody, respectively. The mice were separately injected with 100 µl control (DMEM high sugar medium containing 10% FBS but without PS), MSSA and MRSA supernatants or the neutralized supernatants three times a week, respectively. Animals were monitored every day and the tumor volumes were measured three times a week. Tumor volumes were calculated by the length (L) and the width (W): V = (LW^2^)/2. Animals were sacrificed after a month and all tumor nodules were excised [35].

### Statistical analyses

Comparisons between groups were analyzed by analysis of variance (ANOVA) unless otherwise stated. Adjustment for multiple comparisons between groups was done by LSD test in analysis of variance. The Kruskal Wallis test was applied to analyze the tumor volume. Data are expressed as means ± SE and the results were considered to be statistically significant at *p* < 0.05.

## Results

### MRSA increased the proliferation of OSCC cells

**Figure 1.**
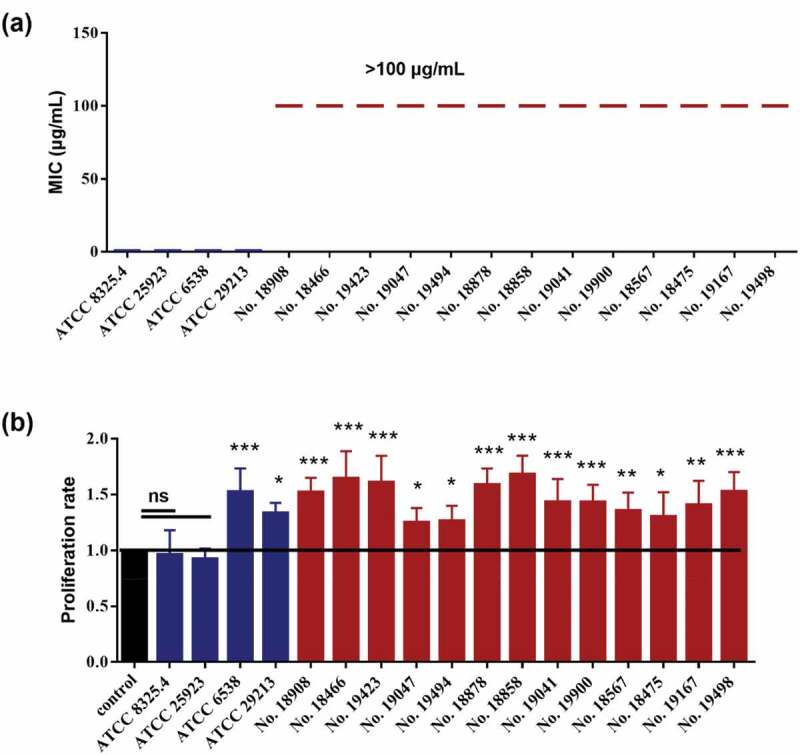
The MICs of *S. aureus* strains and their effects on the proliferation of Cal27 cells. (**a**) The MICs of 17*S. aureus* strains (n = 3). (**b**) The effect of *S. aureus* on the proliferation rates of Cal27 cells (n = 6). *p < 0.05; **p < 0.01; ***p < 0.001. ns, no statistical difference.

Seventeen *S. aureus* strains were employed. We first confirmed their drug-resistant abilities by measuring the MICs to methicillin. As expected, the methicillin MICs of the MSSA strains, including ATCC 25923, ATCC 6538, ATCC 29213 and ATCC 8325.4, were 0.5 µg/ml, while the MICs of the MRSA strains, including No. 18908, No. 18466, No. 19423, No. 19047, No. 19494, No. 18878, No. 18858, No. 19041, No. 19900, No. 18567, No. 18475, No. 19167 and No. 19498, were all higher than 100 µg/ml (Figure 1a). Interestingly, all of the 13 MRSA strains significantly increased the proliferation of Cal27 cells (Figure 1b), while only 2 of 4 MSSA strains could promote the proliferation of Cal27 cells.

### *MRSA promoted the development of OSCC* in vivo

**Figure 2.**
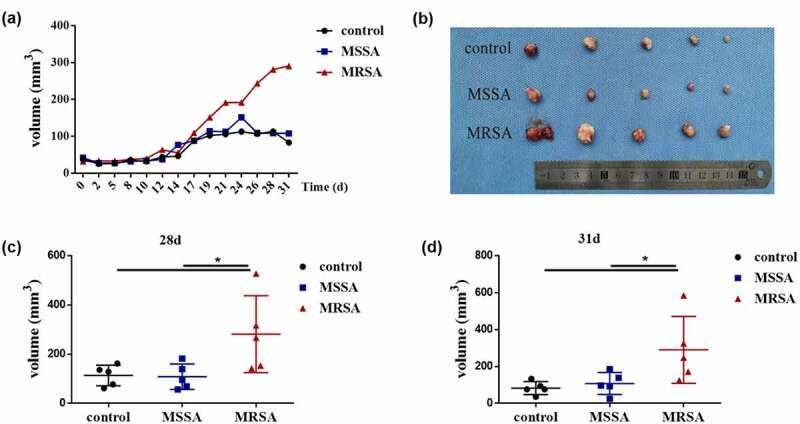
The effects of MRSA and MSSA strains on the development of squamous cell carcinoma *in vivo*. (**a**) Tumor volume in nude mice after the treatment of supernatants from MRSA and MSSA. (**b**) The tumors from MRSA, MSSA and control groups after 31 days. (**c**) Tumor volumes from MRSA, MSSA and control groups after 28 days. (**d**) Tumor volumes from MRSA, MSSA and control groups after 31 days. *p < 0.05 (n = 5).

We then randomly selected ATCC 8325.4 and No. 18466 represented as MSSA and MRSA, respectively, to investigate the relationship between *S. aureus* and OSCC. MRSA significantly increased the cell proliferation of both Cal27 and SCC25, while MSSA had no effects (Figure S1). Then, we investigated the capabilities of MRSA on tumor development *in vivo*. Tumor growth curves indicated that the tumors from the MRSA group grew faster than those from the MSSA and control groups, while there were no differences between MSSA and control groups (Figure 2a). After about 1 month, the tumor size from the MRSA group (290.36 ± 72.44 mm^3^) was significantly larger than that from the MSSA (107.93 ± 23.91 mm^3^) and control (83.13 ± 14.08 mm^3^) groups (Figure 2b-d), indicating the capacity of MRSA to promote the development of OSCC *in vivo*.

### MRSA activated the c-fos and TLR4/NF-κB/MAPK signaling pathways

**Figure 3.**
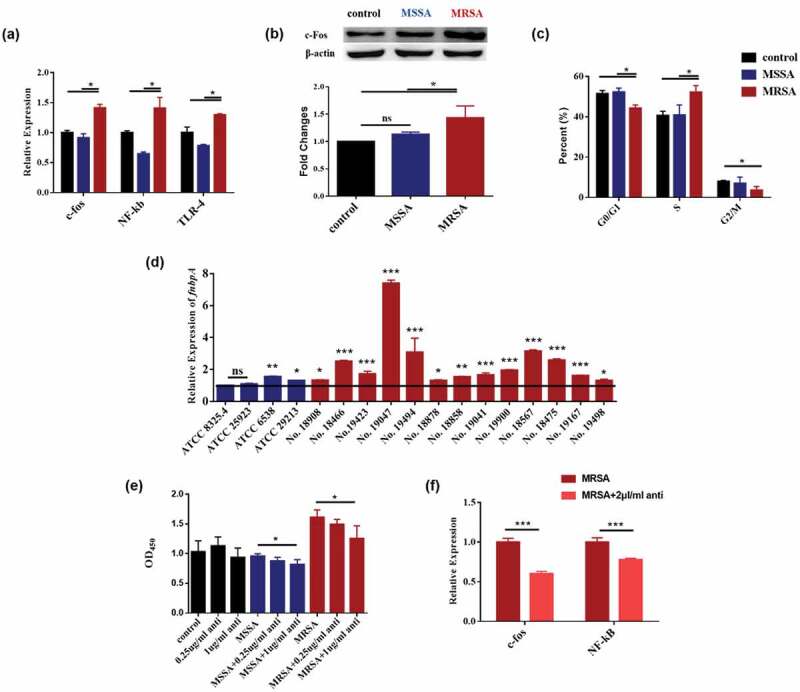
MRSA activated c-fos and TLR4/NF-κB/MAPK signaling pathway by upregulating *fnbpA* and arresting the cell cycles of Cal27 cells. (**a**) The expressions of c-fos, NF-κB and TLR-4 genes of Cal27 cells from MRSA, MSSA and control groups (*n* = 3). (**b**) Protein expression levels of c-fos of Cal27 cells from MRSA, MSSA and control groups (*n* = 3). (**c**) The effects of MRSA and MSSA on cell cycles of cal27 (*n* = 3). *p < 0.05. ns, no statistical difference. (**d**) The expression of *fnbpA* in *S. aureus* strains (*n* = 4). (**e**) FnBPA protein antibody reduced the effects of *S. aureus* strains on Cal27 cell proliferation (*n* = 5). (**f**) FnBPA protein antibody reduced the expression of c-fos and NF-κB genes from Cal27 cells (*n* = 4). *p < 0.05; **p < 0.01; ***p < 0.001. ns, no statistical difference.

To investigate the possible mechanism that MRSA affected the proliferation of OSCC cells, the c-fos and TLR4/NF-κB/MAPK signaling pathways of Cal27 cells were analyzed. The mRNA expression levels of c-fos, and NF-κB TLR-4 were significantly upregulated when treated by MRSA (Figure 3a), while the protein production of c-fos of the cells treated by MRSA was also significantly increased compared to the MSSA and control groups (Figure 3b). When the cells were treated by MRSA, the cell numbers of the G0/G1 phase and G2/M phase were significantly decreased and that of the S phase were significantly increased (Figure 3c), indicating that MRSA stimulated DNA synthesis and cell proliferation.

### *MRSA upregulated* fnbpA *to activate the c-fos and TLR4/NF-κB/MAPK signaling pathways*

Since the FnBPA is one of the key virulence factors in MRSA, we then investgated the relationship between FnBPA and OSCC. The expressions of the *fnbpA* gene were higher in all of 13 MRSA strains and the 2 MSSA strains which can also increase the proliferation of Cal27 cells (Figure 3d) indicating the highly positive correlation between *S. aureus* FnBPA and OSCC cell growth. To confirm the roles of FnBPA, the FnBPA antibody was employed to neutralize the FnBPA protein. The FnBPA antibody had no effects on the proliferation of cells in the control group. However, the antibody significantly decreased the cell proliferations of both Cal27 and SCC25 caused by MRSA (Figure 3e, S2), while the IgG antibody had no significant effects indicating that MRSA enhanced the proliferation of OSCC cells through its FnBPA protein. The FnBPA antibody also significantly decreased the activation of the c-fos and NF-κB caused by MRSA (Figure 3f) indicating that MRSA upregulated the *fnbpA* to activate TLR4/NF-κB/p38 MAPK/c-fos signaling pathway and then to increase the proliferation of tumor cells.

### *Neutralization of FnBPA decreased the promotion of MRSA on OSCC* in vivo

**Figure 4.**
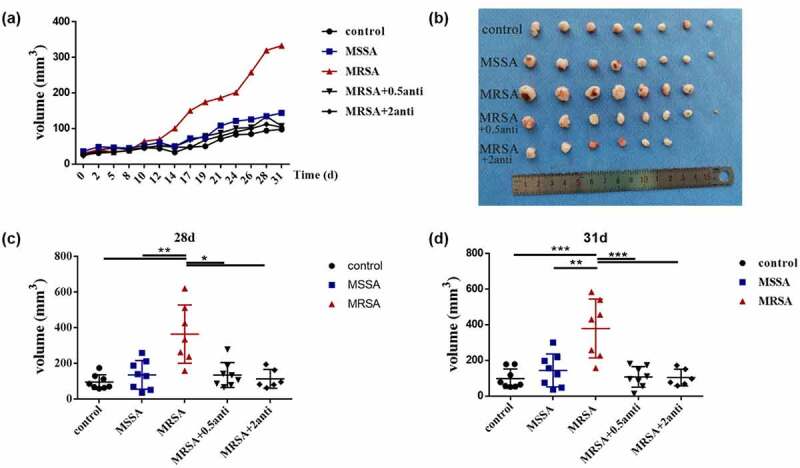
The increased FnBPA protein from MRSA promoted the development of squamous cell carcinoma *in vivo*. (**a**) Tumor volumes after the treatment of control, MSSA, MRSA and MRSA + FnBPA antibody groups. (**b**) The tumors from control, MSSA, MRSA and MRSA + FnBPA antibody groups after 31 days. (**c**) Tumor volumes from control, MSSA, MRSA and MRSA + FnBPA antibody groups after 28 days. (**d**) Tumor volumes from control, MSSA, MRSA and MRSA + FnBPA antibody groups after 31 days. *p < 0.05; **p < 0.01; ***p < 0.001 (*n* = 8, one mouse died accidentally in the MRSA group and two mice died in the MRSA+2 anti group).

We then confirmed the promotion of FnBPA on the development of OSCC *in vivo*. The tumor volume of mice in the anti-FnBPA group was significantly smaller than that of mice in the MRSA group from day 14 and the tumors of mice in the anti-FnBPA group grew more slowly (Figure 4a) similar to the MSSA and control groups. After about 1 month, the tumor size in the MRSA group was significantly larger than that in the MSSA and control groups, while the anti-FnBPA significantly reduced the tumor size (Figure 4b-d), and the tumor size became similar to that of the MSSA and control groups, indicating that FnBPA from MRSA promoted the development of OSCC *in vivo*.

## Discussion

The colonization of MRSA in tumor patients significantly increased their mortality [16]. However, the effect of MRSA on OSCC has not been reported. In this study, we found that MRSA was able to promote tumor proliferation through the increased FnbpA for the first time. The two main FnBPs, FnbpA and FnbpB, from *S. aureus* can not only bind to the FN to invade host tissues [21] but also activate various signaling factors, such as integrin, to promote the pathogenesis including in cancer tissues [36]. The comparative analysis of the expression of the *fnBPA* genes of MRSA and MSSA isolates in our study showed that the *fnbpA* gene was more highly expressed in the MRSA and MSSA strains which can promote the growth of OSCC cells indicating the strong positive correlation between FnBPA and OSCC. We then neutralized the FnBPA and confirmed that the MRSA lost the abilities to promote the growth and development of OSCC both *in vitro* and *in vivo*, which further indicated that the FnBPA was the key factor of the interaction between MRSA and OSCC.

The mammalian cell cycle contains four distinct phases (G0/G1, S, G2 and M), which ensure duplication of genetic material and cell division [37]. Cancer is characterized by uncontrolled proliferation resulting from aberrant activity of various cell cycle proteins, such as CDK4 and CDK6 [38]. Therefore, exploring the cyclic changes of cancer is considered potential targets to judge its proliferation and development prognosis. Numerous studies have found that failure of the G1/S phase and S-phase checkpoints to act properly is particularly deleterious because it may directly elicit chromosomal aberrations and the accumulation of deleterious mutations, which increase the likelihood of the occurrence of cancer [39,40]. In our study, after treatment with MRSA for 24 h, the number of cells in the S phase increased, indicating that MRSA promoted oral squamous cell carcinoma cells into S phase, thereby shortening the time of cell cycle and increasing the proliferation rate.

As a smart pathogen that can infect sterile tissues through open wounds, *S. aureus* expresses a multitude of virulence factors, including adhesive matrix molecules, host cell damage molecules and immunomodulatory molecules. The fibronectin-binding protein FnBPA and FnBPB are the most intensively studied proteins [41]. Both FnBPA and FnBPB proteins contain a signal sequence at the N-terminus and a sorting signal at the C-terminus, but the tandemly arranged motifs of the unstructured region located distal to the A domain of FnBPA is 11, while FnBPB is 10 [20,42]. The *fnbpA* is more likely to be expressed in MRSA strains, while *fnbpB* more likely is expressed in MSSA strains, indicated a direct relationship between drug resistance and virulence, and also suggested the possible different actions with host cells from MRSA and MSSA [43]. Our previous study has confirmed that *fnbpB* expressed by the MSSA strain can induce COX-2 expression and PGE2 production in human oral keratinocyte cells. COX-2 is an enzyme that mediates the synthesis of PGE2, which is involved in the carcinogenesis due to its functions in cell proliferation, invasion, metastasis and angiogenesis. In addition, we found that with the induction of COX-2, expression of the oral cancer-associated genes *cyclin D1* was upregulated and *p16* was downregulated [13]. Different from the FnBPB on host epithelium cells, we found that highly expressed *fnbpA* in MRSA was able to activate the downstream TLR4/NF-κB/p38 MAPK/c-fos pathway, then promoting the proliferation of squamous cell carcinoma, indicating the different actions of FnBPA on cancer cells. Our results also suggested that bacterial resistance can not only affect the response to antibacterial drugs but may also affect its interaction with the host. However, how the FnBPA contributes to the drug resistance is still unknown.

Mounting evidence suggests that FnBPs are involved in host cell signal transduction. The FnBPs of *S. aureus* can significantly inhibit the expression of macrophage fibronectin, while activating the ERK elk1-c-fos, SRC JNK-c-Jun and p38 signaling pathways during the *S. aureus* infection, and the expression of the *fnb* gene can upregulate the c-fos and c-jun in squamous cell carcinoma cells [44,45]. Among these inflammatory factors, C-fos is one of the main subunits of activator protein-1 (AP-1), which is involved in cell proliferation and cell cycle, and decreased after treatment with MAPK inhibitor in cancer cells [46,47]. Although the p38 MAPK can be activated by various pro-inflammatory and stressful stimuli, a previous study demonstrated that p38 MAPK is associated with various cellular responses related to cancer, including proliferation, cell invasion and apoptosis [48–50]. The TLR4 signaling pathway is also activated in a variety of tumor cells to regulate the tumor development [27]. It has been demonstrated that FnBPs can bind to the FNI module of the FN protein, while the FN protein can activate the TLR-4 and then regulate the signaling pathways in a variety of diseases, such as rheumatoid arthritis and chronic skin fibrosis [23–25]. Currently, we found that MRSA can activate the c-fos and TLR4/NF-κB/MAPK signaling pathway, while neutralization of FnBPA significantly decreased the activation indicating that the FnBPA from the MRSA strains can regulating the proliferation of OSCC cells through the TLR4/NF-κB/MAPK signaling pathway. We will investigate the detailed mechanisms on how the FnBPA from MRSA activates the c-fos and TLR4/NF-κB/MAPK signaling pathway in the near future to figure out the whole signaling pathway of OSCC cells responding to the MRSA infection.

There is a strong correlation between the microbiome and tumor development, however, the causation and mechanisms of this correlation remain unclear in most of the cancers [51]. It is generally accepted that the tumor can remodel microbial profiles by creating a more beneficial condition for the shifted microbiome [52–54]. In turn, the microbiome can promote tumorigenesis by establishing an inflammatory environment or through the signal molecule work on host immunity [55]. Antibiotic resistance development is a common feature among many pathogens and tumors [56], and the antibiotics-resistant pathogens have become a major healthcare problem in cancer patients. The development of new therapeutic strategies to simultaneously treat the drug-resistant pathogens and cancer will be a benefit way to treat drug-resistant pathogen-associated cancers. For instance, in addition to killing drug-resistant bacteria, small molecules that modulate antioxidant levels and enhance intracellular ROS could disturb the cellular oxidative environment and induce cancer cell death [57,58]. Meanwhile, the intervention with antibiotics-resistant bacteria can reduce tumor growth and reduce the patient’s immune system damage during the infection process, which could be a new strategy to target tumor and infections.

In summary, we found that MRSA significantly promoted the development of OSCC for the first time. The highly expressed FnBPA protein from MRSA can activate the TLR4/NF-κB/p38 MAPK/c-fos signaling pathway. Our results highlight a potential new strategy to reduce the development of OSCC by targeting the virulence factor of bacteria to block the interaction between drug-resistant pathogens and tumors.

## Supplementary Material

Supplemental Material

## References

[1] Dhanuthai K, Rojanawatsirivej S, Thosaporn W, et al. Oral cancer: a multicenter study. Med Oral Patol Oral Cir Buccal. 2018;23(1): E23–E29. 10.4317/medoral.21999 PMC5822535 29274153

[2] Lu YC, Chen YJ, Wang HM, et al. Oncogenic Function and early detection potential of miRNA-10b in oral cancer as identified by microRNA profiling. Cancer Prev Res (Phila). 2012;5(4):665–10. 22318752 10.1158/1940-6207.CAPR-11-0358

[3] Wen L, Mu W, Lu H, et al. Porphyromonas gingivalis promotes oral squamous cell carcinoma progression in an immune microenvironment. J Dent Res. 2020;99(6):666–675. 32298192 10.1177/0022034520909312

[4] Sasahira T, Kurihara M, Bhawal UK. Sasahira T, Kurihara M, Bhawal UK, et al. Downregulation of miR-126 induces angiogenesis and lymphangiogenesis by activation of VEGF-A in oral cancer. Br J Cancer. 2012;107(4):700–706. 22836510 10.1038/bjc.2012.330 PMC3419968

[5] Irani S. Distant metastasis from oral cancer: a review and molecular biologic aspects. J Int Soc Prev Community Dent. 2016;6(4):265–271. 27583211 10.4103/2231-0762.186805 PMC4981925

[6] Legge CJ, Colley HE, Lawson MA, et al. Targeted magnetic nanoparticle hyperthermia for the treatment of oral cancer. J Oral Pathol Med. 2019;48(9):803–809. 31309616 10.1111/jop.12921

[7] Stashenko P, Yost S, Choi Y, et al. The oral mouse microbiome promotes tumorigenesis in oral squamous cell carcinoma. mSystems. 2019;4(4):e00323–19. 10.1128/mSystems.00323-19 PMC6687944 31387932

[8] McCormack MG, Smith AJ, Akram AN, et al. *Staphylococcus aureus* and the oral cavity: an overlooked source of carriage and infection? Am J Infect Control. 2015;43(1):35–37. 25564121 10.1016/j.ajic.2014.09.015

[9] Panghal M, Kaushal V, Kadayan S, et al. Incidence and risk factors for infection in oral cancer patients undergoing different treatments protocols. BMC Oral Health. 2012;12:22. 22817766 10.1186/1472-6831-12-22 PMC3499184

[10] Hattar K, Reinert CP, Sibelius U, et al. Lipoteichoic acids from *Staphylococcus aureus* stimulate proliferation of human non-small-cell lung cancer cells in vitro. Cancer Immunol Immunother. 2017;66(6):799–809. 28314957 10.1007/s00262-017-1980-4 PMC5445152

[11] Liu T, Li L, Yin L, et al. Superantigen staphylococcal enterotoxin C1 inhibits the growth of bladder cancer. Biosci Biotechnol Biochem. 2017;81(9):1741–1746. 28715277 10.1080/09168451.2017.1350564

[12] Wang YX, Liu SY, Li BL, et al. *Staphylococcus aureus* induces COX-2-dependent proliferation and malignant transformation in oral keratinocytes. J Oral Microbiol. 2019;11(1):1643205. 31448061 10.1080/20002297.2019.1643205 PMC6691923

[13] Wang Y, Ren B, Zhou X, et al. Growth and adherence of *Staphylococcus aureus* were enhanced through the PGE2 produced by the activated COX-2/PGE2 pathway of infected oral epithelial cells. PloS one. 2017;12(5):e0177166. 28472126 10.1371/journal.pone.0177166 PMC5417706

[14] Methicillin-Resistant BM. Staphylococci. J Clin Pathol. 1961;14(4):385. 13686776 10.1136/jcp.14.4.385 PMC480239

[15] Lakhundi S, Zhang K. Methicillin-Resistant *Staphylococcus aureus*: molecular characterization, evolution, and epidemiology. Clin Microbiol Rev. 2018;31(4):e00020–18. 30209034 10.1128/CMR.00020-18 PMC6148192

[16] Hanberger H, Walther S, Leone M, et al. Increased mortality associated with methicillin-resistant *Staphylococcus aureus* (MRSA) infection in the intensive care unit: results from the EPIC II study. Int J Antimicrob Agents. 2011;38(4):331–335. 21798720 10.1016/j.ijantimicag.2011.05.013

[17] Miyake M, Ohbayashi Y, Iwasaki A, et al. Risk factors for methicillin-resistant *Staphylococcus aureus* (MRSA) and use of a nasal mupirocin ointment in oral cancer inpatients. J Oral Maxillofac Surg. 2007;65(11):2159–2163. 17954308 10.1016/j.joms.2007.04.026

[18] Yamashita K, Ohara M, Kojima T, et al. Prevalence of drug-resistant opportunistic microorganisms in oral cavity after treatment for oral cancer. J Oral Sci. 2013;55(2):145–155. 23748454 10.2334/josnusd.55.145

[19] Avery CM, Gandhi N, Peel D, et al. Indications and outcomes for 100 patients managed with a pectoralis major flap within a UK maxillofacial unit. Int J Oral Maxillofac Surg. 2014;43(5):546–554. 24220666 10.1016/j.ijom.2013.10.009

[20] Foster TJ. The remarkably multifunctional fibronectin binding proteins of *Staphylococcus aureus*. Eur J Clin Microbiol Infect Dis. 2016;35(12):1923–1931. 27604831 10.1007/s10096-016-2763-0

[21] Tan X, Coureuil M, Charbit A, et al. Multitasking actors of *Staphylococcus aureus* metabolism and virulence. Trends Microbiol. 2020;28(1):6–9. 31753539 10.1016/j.tim.2019.10.013

[22] Yang X, Qian S, Yao K, et al. Multiresistant ST59-SCC mec IV-t437 clone with strong biofilm-forming capacity was identified predominantly in MRSA isolated from Chinese children. BMC Infect Dis. 2017;17(1):733. 29178841 10.1186/s12879-017-2833-7 PMC5702180

[23] Sofat N, Robertson SD, Wait R. Fibronectin III 13-14 domains induce joint damage via toll-like receptor 4 activation and synergize with interleukin-1 and tumour necrosis factor. J Innate Immun. 2012;4(1):69–79. 21997473 10.1159/000329632 PMC3250657

[24] Bhattacharyya S, Tamaki Z, Wang WX, et al. Fibronectin(EDA) promotes chronic cutaneous fibrosis through toll-like receptor signaling. Sci Transl Med. 2014;6(232):232ra50. 10.1126/scitranslmed.3008264 PMC4414050 24739758

[25] Gondokaryono SP, Ushio H, Niyonsaba F, et al. The extra domain A of fibronectin stimulates murine mast cells via Toll-like receptor 4. J Leukoc Biol. 2007;82(3):657–665. 17575266 10.1189/jlb.1206730

[26] Bai LX, Mao R, Wang JT, et al. ERK1/2 promoted proliferation and inhibited apoptosis of human cervical cancer cells and regulated the expression of c-Fos and c-Jun proteins. Med Oncol. 2015;32(3):57. 25647783 10.1007/s12032-015-0490-5

[27] Wang G, Xu B, Shi F, et al. Protective effect of methane-rich saline on acetic acid-induced ulcerative colitis via blocking the TLR4/NF-kappaB/MAPK pathway and promoting IL-10/JAK1/STAT3-mediated anti-inflammatory response. Oxid Med Cell Longev. 2019;2019:7850324. 31182999 10.1155/2019/7850324 PMC6512011

[28] Jiang XH, Lv B, Wang Y, et al. Bactericidal mechanisms and effector targets of TiO2 and Ag-TiO2 against *Staphylococcus aureus*. J Med Microbiol. 2017;66(4):440–446. 28463658 10.1099/jmm.0.000457 PMC5817198

[29] Guo H, Ren B, Dai H, et al. Reversal of methicillin resistance in *Staphylococcus aureus* by the anthelmintic avermectin. Int J Antimicrob Agents. 2014;44(3):274–276. 25123810 10.1016/j.ijantimicag.2014.05.002

[30] Wu J, Li L, Wang Y, et al. The HSP 90/Akt pathway may mediate artemether‐induced apoptosis of Cal27 cells. FEBS Open Bio. 2019;9(10):1726–1733. 10.1002/2211-5463.12711 PMC6768108 31376209

[31] Zhuo ZJ, Xiao MJ, Lin HR, et al. Novel betulin derivative induces anti-proliferative activity by G2/M phase cell cycle arrest and apoptosis in Huh7 cells. Oncol Lett. 2018;15(2):2097–2104. 29434911 10.3892/ol.2017.7575 PMC5776954

[32] Wang Z, Zhou Y, Han Q, et al. Synonymous point mutation of gtfB gene caused by therapeutic X-rays exposure reduced the biofilm formation and cariogenic abilities of *Streptococcus mutans*. Cell Biosci. 2021;11(1):91. 34001238 10.1186/s13578-021-00608-2 PMC8130306

[33] Zhou Y, Yang H, Zhou X, et al. Lovastatin synergizes with itraconazole against planktonic cells and biofilms of *Candida albicans* through the regulation on ergosterol biosynthesis pathway. Appl Microbiol Biotechnol. 2018;102(12):5255–5264. 29691631 10.1007/s00253-018-8959-8

[34] Percie du Sert N, Hurst V, Ahluwalia A, et al. The ARRIVE guidelines 2.0: updated guidelines for reporting animal research. PLoS Biol. 2020;18(7):e3000410. 32663219 10.1371/journal.pbio.3000410 PMC7360023

[35] Lin NN, Wang P, Zhao D, et al. Significance of oral cancer‐associated fibroblasts in angiogenesis, lymphangiogenesis, and tumor invasion in oral squamous cell carcinoma. J Oral Pathol Med. 2017;46(1):21–30. 27229731 10.1111/jop.12452

[36] Cataldi A, Nascimento AL, Acosta A, et al. Research on bacterial virulence in the developing countries. Biomed Res Int. 2015;2015:972187. 25713813 10.1155/2015/972187 PMC4332757

[37] Otto T, Sicinski P. Cell cycle proteins as promising targets in cancer therapy. Nat Rev Cancer. 2017;17(2):93–115. 28127048 10.1038/nrc.2016.138 PMC5345933

[38] Anders L, Ke N, Hydbring P, et al. A systematic screen for CDK4/6 substrates links FOXM1 phosphorylation to senescence suppression in cancer cells. Cancer Cell. 2011;20(5):620–634. 22094256 10.1016/j.ccr.2011.10.001 PMC3237683

[39] Bartek J, Falck J, Lukas J. CHK2 kinase–a busy messenger. Nat Rev Mol Cell Biol. 2001;2(12):877–886. 11733767 10.1038/35103059

[40] Osborn AJ, Elledge SJ, Zou L. Checking on the fork: the DNA-replication stress-response pathway. Trends Cell Biol. 2002;12(11):509–516. 12446112 10.1016/s0962-8924(02)02380-2

[41] Gries CM, Biddle T, Bose JL, et al. *Staphylococcus aureus* fibronectin binding protein a mediates biofilm development and infection. Infect Immun. 2020;88(5):e00859–19. 32041788 10.1128/IAI.00859-19 PMC7171244

[42] Speziale P, Pietrocola G. The multivalent role of fibronectin-binding proteins A and B (FnBPA and FnBPB) of *Staphylococcus aureus* in host infections. Front Microbiol. 2020;11:2054. 32983039 10.3389/fmicb.2020.02054 PMC7480013

[43] Peacock SJ, Day NPJ, Thomas MG. Peacock SJ, Day NP, Thomas MG, et al. Clinical isolates of *Staphylococcus aureus* exhibit diversity in fnb genes and adhesion to human fibronectin. J Infect. 2000;41(1):23–31. 10942636 10.1053/jinf.2000.0657

[44] Chen HY, Lin MH, Chen CC, et al. The expression of fibronectin is significantly suppressed in macrophages to exert a protective effect against *Staphylococcus aureus* infection. BMC Microbiol. 2017 17; 17(1): 92. 28407745 10.1186/s12866-017-1003-9 PMC5390343

[45] Hayashi Y, Shino Y, Saito K, et al. c-Jun-mediated repression and transactivation of fibronectin. Mol Med Rep. 2008;1(1):99–103. 21479385

[46] Muhammad N, Bhattacharya S, Steele R, et al. Involvement of c-Fos in the promotion of cancer stem-like cell properties in head and neck squamous cell carcinoma. Clin Cancer Res. 2017;23(12):3120–3128. 27965308 10.1158/1078-0432.CCR-16-2811 PMC5468504

[47] Han XR, Zha Z, Yuan HX, et al. KDM2B/FBXL10 targets c-Fos for ubiquitylation and degradation in response to mitogenic stimulation. Oncogene. 2016;35(32):4179–4190. 10.1038/onc.2015.482 PMC4931990 26725323

[48] Nemoto T, Kubota S, Ishida H, et al. Ornithine decarboxylase, mitogen-activated protein kinase and matrix metalloproteinase-2 expressions in human colon tumors. World J Gastroenterol. 2005;11(20):3065–3069. 15918191 10.3748/wjg.v11.i20.3065 PMC4305841

[49] Shin I, Kim S, Song H, et al. H-Ras-specific activation of Rac-MKK3/6-p38 pathway: its critical role in invasion and migration of breast epithelial cells. J Biol Chem. 2005;280(15):14675–14683. 15677464 10.1074/jbc.M411625200

[50] Song H, Ki SH, Kim SG, et al. Activating transcription factor 2 mediates matrix metalloproteinase-2 transcriptional activation induced by p38 in breast epithelial cells. Cancer Res. 2006;66(21):10487–10496. 17079470 10.1158/0008-5472.CAN-06-1461

[51] Zong Y, Zhou Y, Liao B, et al. The interaction between the microbiome and tumors. Front Cell Infect Microbiol. 2021;11:673724. 34532297 10.3389/fcimb.2021.673724 PMC8438519

[52] Mima K, Sukawa Y, Nishihara R, et al. Fusobacterium nucleatum and T cells in colorectal carcinoma. JAMA Oncol. 2015;1(5):653–661. 26181352 10.1001/jamaoncol.2015.1377 PMC4537376

[53] Burns MB, Blekhman R. Integrating tumor genomics into studies of the microbiome in colorectal cancer. Gut Microbes. 2019;10(4):547–552. 30556775 10.1080/19490976.2018.1549421 PMC6748619

[54] Burns MB, Montassier E, Abrahante J, et al. Colorectal cancer mutational profiles correlate with defined microbial communities in the tumor microenvironment. PLoS Genet. 2018;14(6):e1007376. 29924794 10.1371/journal.pgen.1007376 PMC6028121

[55] Manzoor SS, Doedens A, Burns MB. The promise and challenge of cancer microbiome research. Genome Biol. 2020;21(1):131. 32487228 10.1186/s13059-020-02037-9 PMC7265652

[56] Avner BS, Fialho AM, Chakrabarty AM. Overcoming drug resistance in multi-drug resistant cancers and microorganisms: a conceptual framework. Bioengineered. 2012;3(5):262–270. 22750915 10.4161/bioe.21130 PMC3477693

[57] Dharmaraja AT. Role of reactive oxygen species (ROS) in therapeutics and drug resistance in cancer and bacteria. J Med Chem. 2017;60(8):3221–3240. 28135088 10.1021/acs.jmedchem.6b01243

[58] Yoo MH, Xu XM, Carlson BA, et al. Thioredoxin reductase 1 deficiency reverses tumor phenotype and tumorigenicity of lung carcinoma cells. J Biol Chem. 2006;281(19):13005–13008. 16565519 10.1074/jbc.C600012200

