# Supplementary material for: The FnBPA from methicillin-resistant Staphylococcus aureus promoted development of oral squamous cell carcinoma
Source: J Oral Microbiol. 2022 Jul 15;14(1):2098644. doi: 10.1080/20002297.2022.2098644 (PMC9291692; doi:10.1080/20002297.2022.2098644)
Supplement: Supplemental Material [file ZJOM_A_2098644_SM5803.docx]

**Table S**[**1**](https://link.springer.com/article/10.1007/s00253-018-8959-8#MOESM1)**.** Primers used in this study

| **Genes** | **Primers** |
| --- | --- |
| *TLR-4* | For 5'- GCCCTGCGTGGAGGTGGTTC-3' |
|  | Rev 5'- TGAGAAGGGGAGGTTGTCGGGG-3' |
| *c-fos*  *NF-**кb*  *GAPDH* | For 5'- CCGGGGATAGCCTCTCTTACT-3'  Rev 5'- CCAGGTCCGTGCAGAAGTC-3'  For 5'-TGGGGACTACGACCTGAATG-3'  Rev 5'-GGGGGCACGATTGTCAAAGA-3'  For 5'-GTCTTCACTACCATGGAGAAGG-3' |
|  | Rev 5'-TCATGGATGACCTTGGCCAG-3' |
| *fnbpA* | For 5′-AAACAATAGAAGAAACGGATTCATC -3′ |
|  | Rev 5′-TCAATTGGATTTGATTCCTCAGA -3′ |
| *16S* | For 5′-TCCGGAATTATTGGGCGTAA -3′  Rev 5′-CCACTTTCCTCTTCTGCACATCA -3′ |

**The protocol of IgG antibody treatment**

The protocol of IgG antibody treatment was similar to that of FnBPA antibodies. *S. aureus* was cultured overnight at 37 ^o^C in TSB medium and then diluted in fresh TSB medium at 1:5 to incubate at 37 ^o^C for 2 h. Then the bacteria were harvested by centrifugation (4,000 g, 4 °C, 10 min), washed with phosphate buffer saline and re-suspended (1 × 10^8^ CFU / ml) in high glucose DMEM Medium with 10% FBS. The IgG antibody (1μg/mL, Beyotime, China) were added into the *S. aureus* suspension and incubated at 37 ^o^C for 2 h. Then the filtrated supernatants were used to treat the cells as described in manuscript.

**
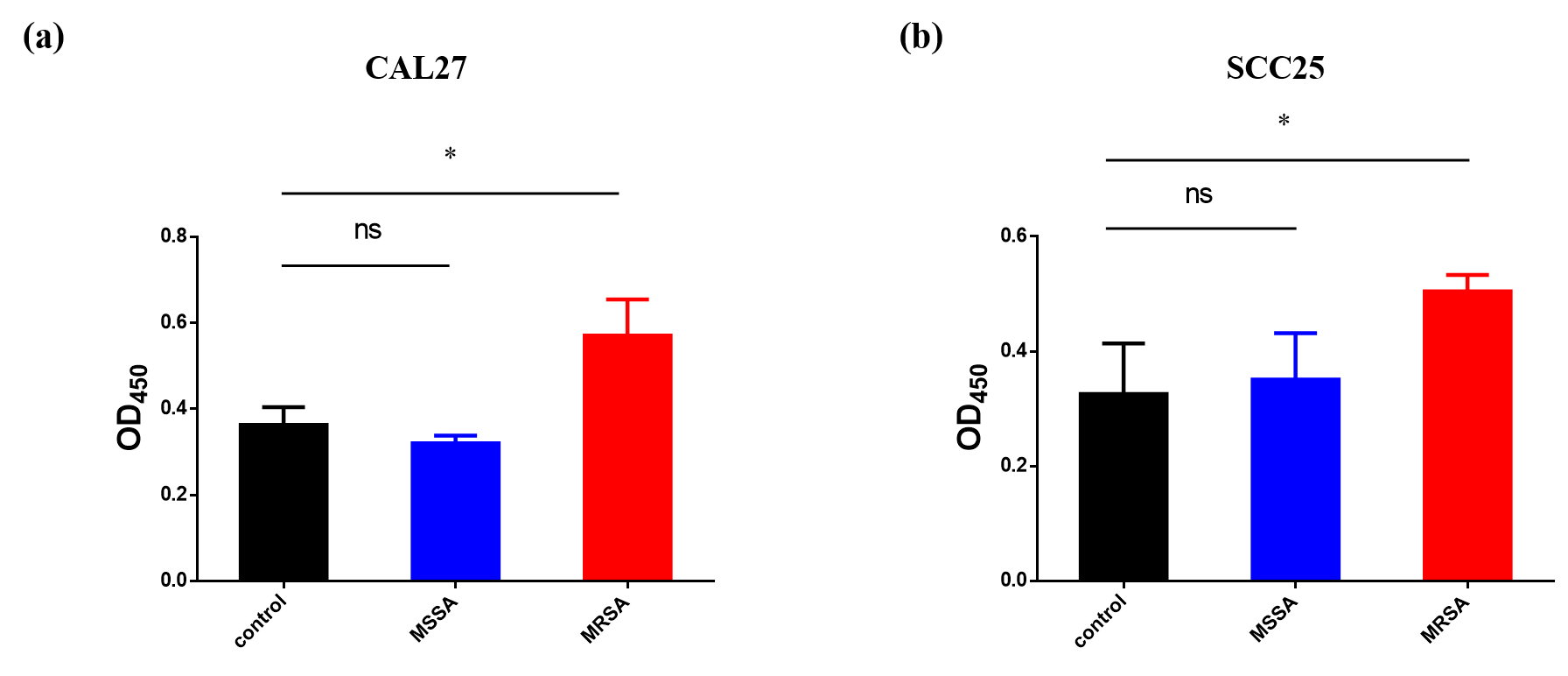
**

**Figure S1**. Proliferation of OSCC cells treated by *S. aureus*. (**a**) Cal27 cells. (**b**) SCC25 cells.

**
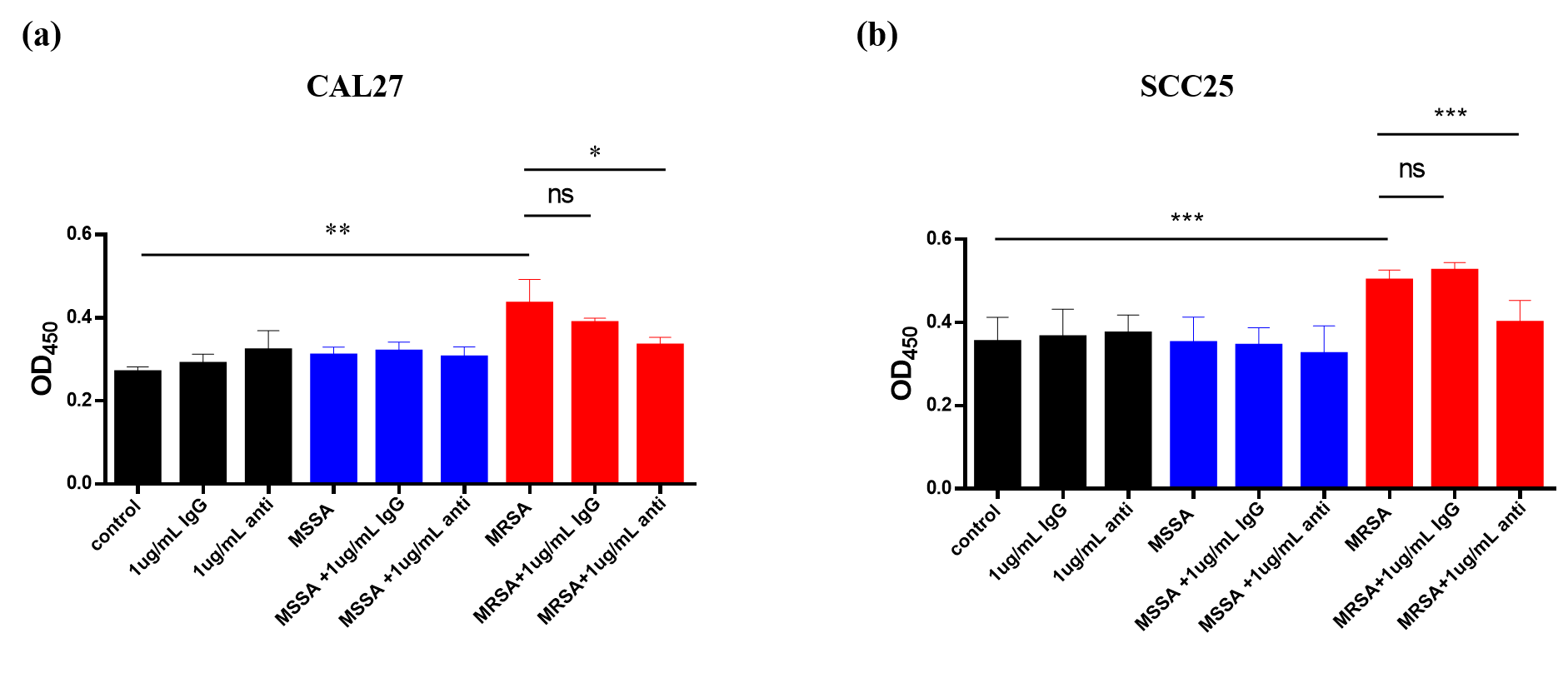
**

**Figure S2**. Proliferation of OSCC cells treated by MRSA, IgG antibody and FnBPA protein antibody. (**a**) Cal27 cells. (**b**) SCC25 cells.
